# Isolation of a Bis-Iodurated Tetra-THF as a Trace Product from the Oxidation of Squalene with RuO_4_ and Its Double Ring Expansion to a Novel bis-THF-bis-THP Compound

**DOI:** 10.3390/molecules16075362

**Published:** 2011-06-27

**Authors:** Vincenzo Piccialli, Sabrina Zaccaria, Roberto Centore, Angela Tuzi, Nicola Borbone, Giorgia Oliviero

**Affiliations:** 1 Dipartimento di Chimica Organica e Biochimica, Università degli Studi di Napoli "Federico II", Via Cynthia 4, 80126, Napoli, Italy; Email: sabrina.zaccaria@unina.it (S.Z.); 2 Dipartimento di Chimica ‘‘Paolo Corradini’’, Università degli Studi di Napoli ‘‘Federico II’’, Via Cinthia 4, 80126, Napoli, Italy; Email: roberto.centore@unina.it (R.C.); angela.tuzi@unina.it (A.T.); 3 Dipartimento di Chimica delle Sostanze Naturali, Università degli Studi di Napoli "Federico II", Via D. Montesano 49, 80131, Napoli, Italy; Email: nicola.borbone@unina.it (N.B.); golivier@unina.it (G.O.)

**Keywords:** ruthenium tetroxide, squalene, poly-THF, tetrahydrofuran, tetrahydropyran, rearrangement-ring expansion

## Abstract

A novel bis-iodurated polyether compound, based on an unprecedented tetra-THF backbone, has been isolated as a trace by-product of the oxidation of squalene with the catalytic system RuO_2_(cat.)/NaIO_4_. The double *erythro* configuration of the central portion of the molecule furnishes the first indirect support of the previously postulated pathway operating in the oxidative pentacyclization of the isoprenoid substrate. A bidirectional double oxidative bis-cyclization is invoked to explain the formation of this compound. The isolated substance was successfully subjected to a double rearrangement-ring expansion to give a novel bis-THF-bis-THP compound.

## 1. Introduction

**Scheme 1:**
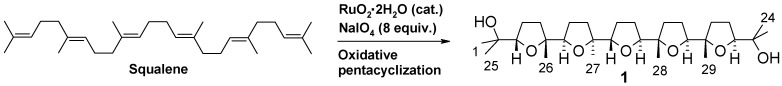
Stereoselective synthesis of a pentacyclic poly-THF (**1**) by RuO_4_-catalysed oxidative polycyclization of squalene.

Some years ago we discovered a novel cascade process catalysed by RuO_4_ generated *in situ* by the action of NaIO_4_ on RuO_2_, the pre-catalytic species employed to generate RuO_4_ [1]. This is a unique process by which a poly-THF backbone, made up of adjacently linked THF rings, can be built-up in a single step and in a stereoselective manner starting from polyenes characterized by a repetitive 1,5-diene structural motif [2,3,4,5,6,7,8]. In particular, oxidation of squalene gives rise to penta-THF compound **1** (Scheme 1) containing ten stereogenic centres. Previous studies carried out in our group had suggested that steric and chelation control factors concur to determine the stereochemical outcome of the process [6].

**Scheme 2:**
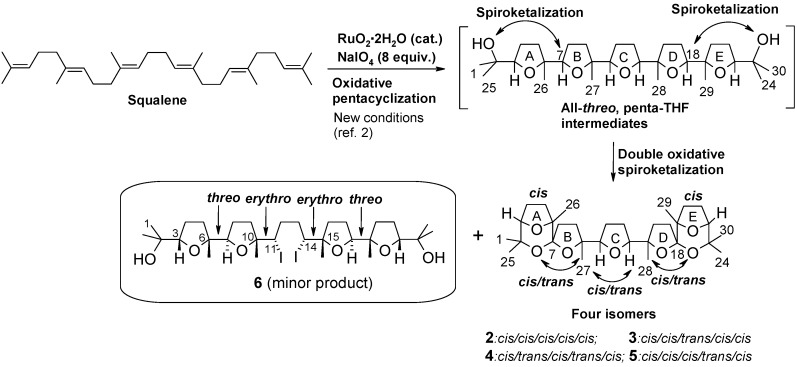
Synthesis of polycyclic polyethers by one-step RuO_4_-catalysed oxidative polycyclization/oxidative spiroketalization of squalene.

In a more recent investigation, the use of different cyclization conditions led to a different stereochemistry of the process [9]. In particular, four new C_30_ isomeric heptacyclic polyether substances (compounds **2**-**5**, Scheme 2) were obtained through a unique seven-step cascade process featuring a pentacyclization of squalene followed by a double oxidative spiroketalization at the two bis-THF termini of the first-formed penta-THF intermediates. Careful HPLC isolation of latter substances for X-ray studies [9] allowed also the isolation of tetra-THF **6** (Scheme 2), a very minor side-product of the process, possessing a C*_S_*-symmetric structure embodying two terminal *cis*-*threo*-*trans* bis-THF moieties connected by a central bis-iodurated tetracarbonious segment. The determination of the stereostructure of compound **6**, the mechanistic implication of its isolation as well as its double rearrangement-ring expansion to a new bis-THP polyether compound, are discussed in the present paper.

## 2. Results and Discussion

### 2.1. Chemistry

**Scheme 3:**
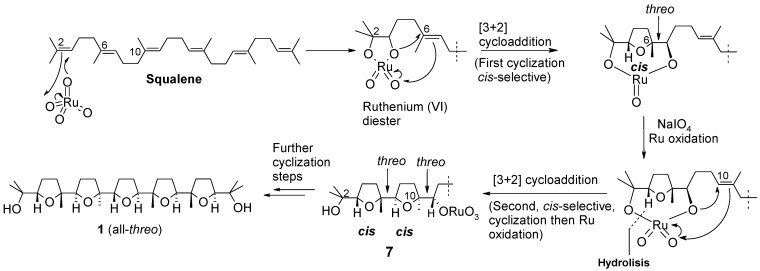
Ru-catalysed cascade sequence leading to penta-THF **1**.

The structure of compound **6** was determined by X-ray diffraction analysis carried out on a single crystal of the substance obtained by slow evaporation of a chloroform solution. The most interesting, and unusual feature of this compound is the double *erythro* configuration around the C10/C11 and C14/C15 bonds. In fact, previous studies from our group had demonstrated that the oxidation of both linear and isoprenoid polyenes constantly furnishes poly-THF compounds possessing *threo* inter-THF relationships. This is consistent with the *syn* addition (a [3+2] cycloaddition) of a O=Ru-O portion across each involved C-C double bond, along the all-*trans* polyene chain (Scheme 3), that also agrees with mechanistic proposals for related oxidative mono-cyclization of 1,5-dienes catalysed by RuO_4_ [10,11,12,13,14,15] and related oxo-species OsO_4_ [16,17,18] and MnO_4_^-^ [19,20,21,22,23,24,25,26,27], as well as rhenium (VII)-mediated oxidative polycyclization of hydroxypolyenes [28,29,30,31].

**Scheme 4:**
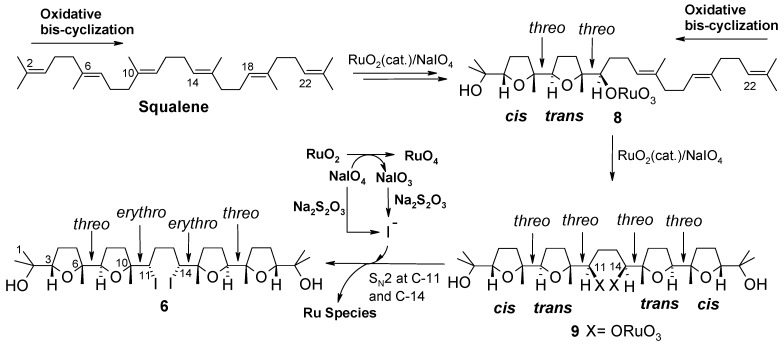
A plausible path for the formation of tetra-THF **6**.

Based on these precedents, formation of **6** was intriguing and could be rationalised through a double *cis*/*trans*-selective oxidative bis-cyclization process (Scheme 4). Each bis-cyclization event involves three consecutive double bonds of the polyene chain starting from the terminal ones. In particular, attack of RuO_4_ to the Δ^2^ double bond induces two successive cyclization steps giving rise to bis-THF intermediate **8**, in the same manner as shown for the synthesis of **1** (see intermediate **7**, Scheme 3). A second bis-cyclization would then occur at the other side of the molecule by attack of RuO_4_ at the terminal, Δ^22^, double bond to give the all-*threo* tetra-THF **9** still possessing two oxoruthenate appendages linked to C-11 and C-14. It can be presumed that a double substitution of the ruthenium-containing portions, with inversion of configuration at involved carbon centres, would then occur during the reductive quenching of the process, by iodide ions probably generated *in situ* by the action of tiosulphate on iodate in turn produced during the oxidation of RuO_2_ to RuO_4_. It cannot be excluded that iodide could originate from reduction of periodate itself not completely consumed in the reaction medium. It is probable that such a side-process could be due to the higher concentration of the reaction medium in the new experimented conditions [9].

In order to enlarge the range of polyether substances accessible through the Ru-mediated polycyclization process we began an exploration of the possible post-synthetic modifications of some of the poly-THF backbones obtained through the above process. We have previously demonstrated that progressive structural simplification of compound **1** to small-sized poly-THF compounds can be achieved *via* an iterative PCC-mediated oxidative cleavage/reduction [7] sequence. The entire process is possible due to the two alcohol functionalities adjacent to the terminal THF rings that are prone to be intercepted by PCC [32]. In addition, a new type of cytotoxic spyroketal poly-THF compound, strictly related to bis-spiroketals **2**-**5**, could be accessed through a PCC-mediated oxidative spiroketalization process starting from **1** [3]. In a more recent study we have also shown that the same oxidant, or the related system PCC-H_5_IO_6_, is able to attack the angular CH position of the THF ring in various mono and poly-THF substrates leading to either the oxidative opening of the THF ring or the oxidative cleavage of suitable inter-THF bonds [33].

**Scheme 5:**
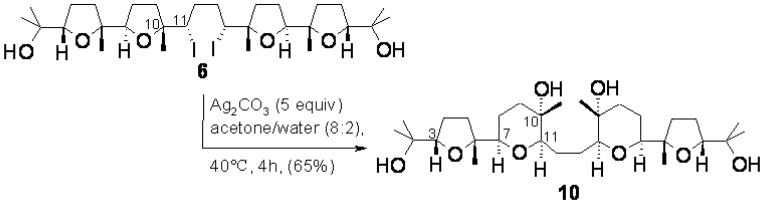
Double rearrangement-ring expansion of compound **6**.

As a continuation of this project, we envisaged that compound **6**, possessing a central bis-(α-iodo-THF) portion, could be a good model compound to probe a double rearrangement-ring expansion process involving the two internal THF rings as a means to access a new type of mixed THF-THP polyether compounds further functionalised for successive synthetic manipulations providing access to new polyether polycyclic materials. This type of reaction has previously been carried out on substances containing a single α-iodo-THF subunit [34] but has never been attempted on a substrate containing two α-iodo-THF moieties and, in particular, as far as we know, the double rearrangement-ring expansion of a bis-THF substance has never been accomplished. Related chemistry has been successfully employed for example in the synthesis of salynomicin [25] as well as in the synthesis of a bis-oxepane portion of hemibrevetoxin [35]. Pleasingly, when compound **6** was reacted with excess Ag_2_CO_3_ (5 equiv.) in acetone/water (8:2, 40 °C, 4 h), compound **10** was obtained in a 65% yield demonstrating the feasibility of the projected transformation (Scheme 5).

Proof for the structure **10** was gained by chemical and high-field 2D-NMR evidence. Attempted acetylation and benzoylation under standard conditions only delivered unreacted **10** indicating, as expected, the presence of tertiary hydroxyl groups in this compound.

A ^1^H^1^H-COSY experiment at 700 MHz indicated the presence in **10** of the two five-proton spin systems H-3/H_2_-4/H_2_-5 and H-7/H_2_-8-H_2_-9 belonging to the two adjacent rings as well as the H-11/H_2_-12 spin system. Assignment of each of these spin systems to the proper ring came from considerations of spectral data and comparison with strictly related THF- and THP-containing substances. In particular, the signals resonating at δ 3.86 and 2.36 were assigned, respectively, to the angular THF proton (H-3) and to the H_α_-5 proton based on the good agreement of their chemical shift values with those typically exhibited by these protons in strictly analogous poly-THF substances including the same *cis*-THF-containing substructure, previously synthesised in our laboratories [1,2,3,4,5,6,7,8]. This deduction suggested that the two higher field one-proton resonances at δ 3.26 (H-7) and 3.16 (H-11) could be ascribable to the angular hydrogens in the THP ring.

The good proton dispersion of the signals in the ^1^H-NMR spectrum of **10** allowed us to fully analyse some crucial signals. In particular, the presence of a THP ring in **10** was corroborated by *J* values (*J* = 12.5, 3.6 Hz) of the H-9 equatorial proton resonating as a clean double triplet at δ 1.89, as expected for an equatorial proton next to a quaternary centre (C-10) in a six-membered ring possessing a chair conformation. In addition, a W coupling observed between the signal at δ 1.54 for H_ax_-9 and the singlet methyl resonance at δ 1.19, ascribable to the C-10 methyl group, also pointed to the presence of a THP ring and the axial nature of that methyl. W-type long-range couplings were also observed between the singlet resonances at δ 1.23 and 1.05 allowing assignment of these signals to the two methyls belonging to the terminal 2-hydroxyisopropyl group. Similarly, a long-range coupling between the methyl signal at δ 1.12 and the H_a_-5 resonance at δ2.36 allowed to assign the former resonance to the angular methyl of the THF ring (C6-Me).

**Figure 1:**
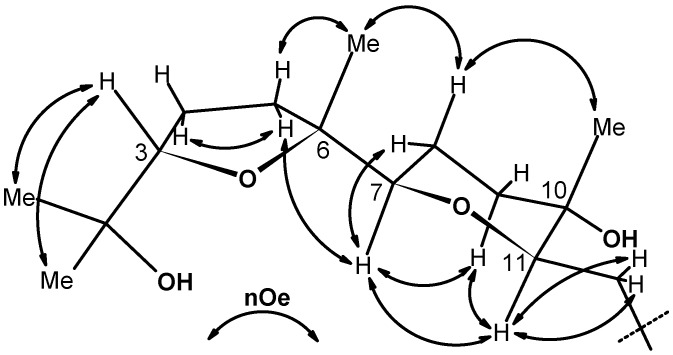
Summary of some significant 700 MHz NOESY correlations for compound **10** (due to the symmetry, half molecule is shown).

These conclusions were reinforced by data from a very informative 700 MHz NOESY experiment (Figure 1) that also provided conclusive information on the relative configuration of the C-7, C-10 and C-11 centres belonging to the THP ring in **10**. In particular, the *cis* nature of the THP ring was inferred by the presence of a strong correlation peak between signals for the H-7 and H-11 angular protons. Similarly, the axial nature of the C-10 methyl, was further corroborated by a nOe correlation between its resonance at δ 1.19 and that of the H_ax_-8 proton at δ 1.74. The rest of nOe cross peaks shown in Figure 1 were in full agreement with the given stereostructure.

### 2.2. X-ray crystallography.

**Figure 2:**
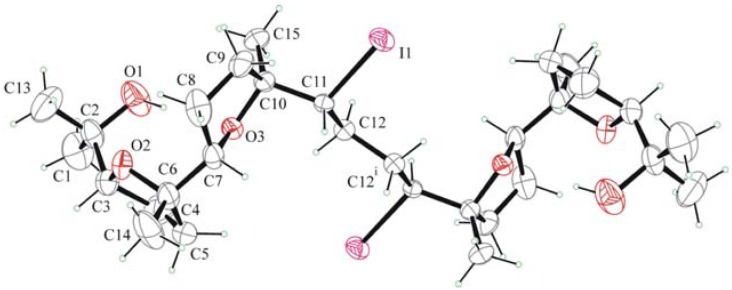
ORTEP view of **6**.

Molecules of **6** in the crystals are centrosymmetric (*C*_i_ point group) as they lie on crystallographic inversion centres (Figure 2). The molecules have a stretched winding shape, which is due to the double *cis*-*trans* sequence of the 2,5-disubstituted THF rings and to the *trans*-planar conformation of the carbon chain. 

The molecular conformation is stabilized by an intramolecular H bonding between O-H donor and the oxygen acceptor of the inner THF ring (O1−H∙∙∙O3 0.983, 2.224, 3.175(9) Å, 163°). Ring puckering coordinates of the inner THF ring are q_2_ = 0.356(6) Å j_2_ = 211(1)°, and of the outer are q_2_ = 0.316(7) Å j_2_ = 324(1)°. On the basis of the calculated phase angles, it can be argued that both are basically in envelope conformation, with C7 and C4 atoms out of the envelope plane.

**Figure 3:**
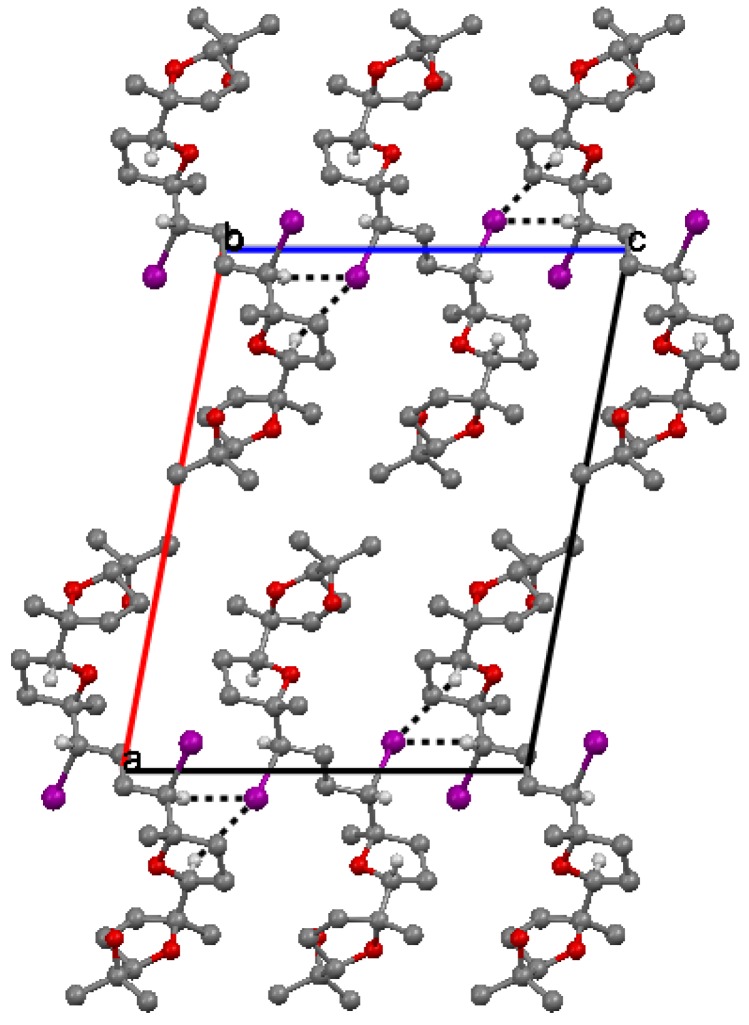
Crystal packing of **6** viewed down **b**.

The packing of molecules is accomplished through weak H bonding interactions between iodine atoms as bifurcated acceptors and methyne C-H donors [36]. This is clearly shown in Figure 3. Chains of H-bonded molecules are formed which run along **b** + **c** and **b** – **c** lattice directions. The weak H-bonding leads to the formation of ring patterns having graph set descriptor 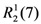. Along **a** molecules are stacked in layers through van der Waals contacts.

## 3. Experimental

### 3.1. General

All reagents were purchased (Aldrich) at the highest commercial quality and used without further purification. Reactions were monitored by thin-layer chromatography carried out on precoated silica gel plates (Merck 60, F_254_, 0.25 mm thick). Merck silica gel (Kieselgel 40, particle size 0.063-0.200 mm) was used for column chromatography. HPLC separations were carried out on a Varian 2510 apparatus equipped with a Waters R403 dual cell differential refractometer using Phenomenex 250 × 10 mm, Phenomenex 250 × 4.6 mm and Nucleosil 250 × 10 mm columns. NMR experiments were performed on Varian Mercury Plus 400 MHz, Varian Unity Inova 700 MHz and Gemini 200 spectrometers in CDCl_3_. Proton chemical shifts were referenced to the residual CHCl_3_ signal (7.26 ppm); ^13^C-NMR chemical shifts were referenced to the solvent (77.0 ppm). J values are in Hz. Abbreviations for signal coupling are as follows: s, singlet; d, doublet; t, triplet; q, quartet; m, multiplet. IR spectra were collected on a Jasco FT-IR-430 spectrometer. High Resolution MS spectra were recorded on a Bruker APEX II FT-ICR mass spectrometer using the electrospray ionization (ESI) technique in positive mode.

### 3.2. Synthesis

Squalene (50 g, 122 mmol) was placed into a 5 L round-bottomed flask equipped with a mechanical stirrer and dissolved in the biphasic mixture EtOAc/CH_3_CN/H_2_O (3:3:2, 1.6 L). The solution was cooled to 0 °C and NaIO_4_ (8 equiv., 976 mmol, 209 g) and RuO_2_•2H_2_O (20 mol%, 24.4 mmol, 3.25 g) were sequentially added under vigorous stirring. After 30 min excess Na_2_S_2_O_3_•5H_2_O was added and the mixture was stirred for further 10 min and then filtered through a Buchner funnel. The solid left on the Buchner was thoroughly washed with EtOAc and the resulting biphasic solution was concentrated *in vacuo*. The aqueous suspension was extracted with EtOAc (3 × 300 mL). The combined organic phase was dried (Na_2_SO_4_) and evaporated *in vacuo* to give an oily product that was chromatographed on silica gel (50 × 8 cm column) eluting with petroleum ether (40-70)/Et_2_O mixtures (from 7:3 to 100% ether) and then with CHCl_3_/MeOH mixtures (up to CHCl_3_/MeOH 8:2) to give three fractions: fraction A (7.40 g) eluted before penta-THF **1**; fraction B (4.75 g) containing penta–THF **1** and fraction C (35.18 g) eluted after penta-THF **1**. A sample (500 mg) of the less polar fraction A was separated by HPLC (250×10 mm column, eluent: hexane-EtOAc, 8:2, flow 2.5 mL/min) to give previously isolated bis-spiroketals **2-5**. The fraction eluted in the range 20-30 min was subjected to a further reversed-phase HPLC separation (250×10 mm column; flow: 1.0 mL/min, eluent: MeOH/H_2_O, 8:2, *t*_R _= 14.5 min) to give pure *2,2'-(5',5''-(1,4-diiodobutane-1,4-diyl)bis(2,5'-dimethyl-octahydro-2,2'-bifuran-5',5-diyl))dipropan-2-ol *(**6**, 2.5 mg, 0.03%). IR (neat): υ_max_ 3706, 3780, 1054, 1013 cm^-1^; ^1^H-NMR: (400 MHz, CDCl_3_) d 4.00 (1H, bd, *J *= 9.7), 3.91 (1H, m), 3.85 (1H, dd, *J *= 7.7, 5.2), 2.32 (1H, m), 2.23 (1H, ddd, *J *= 12.1, 8.7, 8.7), 1.45, 1.25, 1.13, 1.09 (3H each, s’s, 4xMe); ^13^C-NMR (50 MHz, CDCl_3_): d 85.6, 85.0, 84.4, 83.0, 71.9, 47.9, 39.9, 36.4, 34.7, 27.9, 27.2, 25.8, 24.9, 24.3, 22.9; HRMS (ESI) *m/z* calcd for C_30_H_52_I_2_NaO_6_ [M+Na]^+^ 785.1751, found 785.1748.

### 3.3. Ring expansion of ***6*** to ***10***

To compound **6** (1.5 mg, 0.02 mmol) dissolved in acetone-water (4:1, 500 μL) was added silver carbonate (16.8 mg, 0.1 mmol) and the mixture stirred at 40 °C. After 4h, the mixture was filtered and the solid thoroughly washed with acetone. The organic phase was taken to dryness to give an oily product. HPLC purification (250 × 4.6 mm column; flow: 1.0 mL/min; CHCl_3_MeOH, 98:2) gave pure *2,2'-(butane-1,4-diyl)bis(6-(5-(2-hydroxypropan-2-yl)-2-methyl-tetrahydrofuran-2-yl)-3-methyl-*

*tetrahydro-2H-pyran-3-ol)* (**10,** 0.7 mg, 65%, *t*_R_ =16.5 min). Oil; IR (neat): υ_max_ 3440 cm^-1^; ^1^H-NMR: (700 MHz, CDCl_3_) d 3.86 (1H, dd, *J* = 8.4, 3.7), 3.26 (1H, bdd, *J* = 7.0, 7.0), 3.16 (1H, bd, *J* = 6.3), 2.36 (1H, ddd, *J* = 10.0, 10.0, 10.0), 2.05 (1H, dddd, *J* = 12.7, 9.6, 3.7, 3.7), 2.03-1.94 (2H, m), 1.89 (1H, ddd, *J* = 12.5, 3.6, 3.6), 1.75 (2H, m), 1.54 (2H, m), 1.31 (1H, m), 1.23, 1.19, 1.12, 1.05 (3H each, s’s, 4 × Me); HRMS (ESI) *m/z* calcd for C_30_H_54_NaO_8_ [M+Na]^+^ 565.3716, found 565.3710.

### 3.4. X-Ray Crystallography

Crystals of **6** suitable for X-ray analysis were obtained from CHCl_3_ by slow evaporation of the solvent. Data were collected at 298 K on a Bruker-Nonius Kappa-CCD diffractometer using graphite monochromated MoK*α* radiation (*λ* = 0.71073 Å). Data reduction and multi-scan absorption correction were done using SADABS program [37]. The structure was solved by direct methods (SIR97 program [38]) and refined by the full matrix least-squares method on *F*^2^ using SHELXL-97 program [39] with the aid of the program WinGX [40]. Non-hydrogen atoms were refined anisotropically. H atoms of the hydroxy group was located in difference Fourier maps and refined with U_iso_ = 1.2∙U_eq_ of the carrier atom. The positions of the other H atoms were determined stereochemically and refined by the riding model with U_iso_ = 1.2∙U_eq_ of the carrier atom (1.5∙U_eq_ for H atoms of methyl groups). Ring puckering coordinates [41] were determined using the program PARST [42]. The analysis of the crystal packing and the drawing of the molecule were performed using the programs Mercury [43] and ORTEP [44]. Crystal and refinement data are summarized in Table 1. CCDC reference number 821334 contains the supplementary crystallographic data for **6**.

## 4. Conclusion

In conclusion, the isolation of bis-iodocompound **6** was interesting from both a mechanistic and a synthetic point of view. Its existence among the oxidation products of squalene with RuO_4_ was indicative of the existence of an intermediate species (see **9**, Scheme 4), carrying oxoruthenium substituents, likely ORuO_3_ groups, adjacent to the two internal THF rings, able to undergo a facile nucleophilic displacement, that enforces the mechanistic hypothesis previously put forward to explain the formation of penta-THF **1** from the same substrate (Scheme 3). In addition, the postulated mechanism for the formation of **6** also suggests a new possible use of the RuO_4_-catalysed polycyclization process where suitable polyenes can be induced to undergo bidirectional poly-THF-forming oxidative sequences. The facile access to a novel type of bis-THF-bis-THP compound (**10**) has been demonstrated *via* a double ring-enlargement process. Studies are in progress to further develop the chemistry presented here toward the synthesis of new THP-containing polyether compounds.

## References

[1] Bifulco G., Caserta T., Gomez-Paloma L., Piccialli V. (2002). RuO_4_-promoted *syn*-oxidative polycyclization of isoprenoid polyenes: a new stereoselective cascade process. Tetrahedron Lett..

[2] Centore R., Tuzi A., Zaccaria S., Piccialli V. Synthesis, stereostructure and H-bonding patterns of a tris-THF compound. J. Chem. Crystallogr..

[3] Piccialli V., Oliviero G., Borbone N., Tuzi A., Centore R., Hemminki A., Ugolini M., Cerullo V. (2009). Discovery of a new PCC-mediated stereoselective oxidative spiroketalization process. An access to a new type of poly-THF spiroketal compound displaying anticancer activity. Org. Biomol. Chem..

[4] Piccialli V., Borbone N., Oliviero G. (2008). RuO_4_-catalyzed oxidative polycyclization of the C*_S_*-symmetric isoprenoid polyene digeranyl. An unexpected stereochemical outcome. Tetrahedron.

[5] Piccialli V. (2007). Oxidative cyclization of dienes and polyenes mediated by transition-metal-oxo species. Synthesis.

[6] Piccialli V., Caserta T., Caruso L., Gomez-Paloma L., Bifulco G. (2006). RuO_4_-mediated oxidative polycyclization of linear polyenes. A new approach to the synthesis of the bis-THF diol core of antitumour *cis-cis* adjacent bis-THF annonaceous acetogenins. Tetrahedron.

[7] Caserta T., Piccialli V., Gomez-Paloma L., Bifulco G. (2005). RuO_4_-catalyzed oxidative polycyclization of squalene. Determination of the configuration of the penta-tetrahydrofuranyl diol product. Tetrahedron.

[8] Bifulco G., Caserta T., Gomez-Paloma L., Piccialli V. (2003). RuO_4_-promoted oxidative polycyclization of isoprenoid polyenes. A further insight into the stereochemistry of the process. Tetrahedron Lett..

[9] Piccialli V., Zaccaria S., Borbone N., Oliviero G., D'Errico S., Hemminki A., Cerullo V., Romano V., Tuzi A., Centore R. (2010). Discovery of a novel one-step RuO_4_-catalysed tandem oxidative polycyclization/double spiroketalization process. Access to a new type of polyether bis-spiroketal compound displaying antitumour activity. Tetrahedron.

[10] Carlsen P.H.J., Katsuki T., Martin V.S., Sharpless K.B. (1981). A greatly improved procedure for ruthenium tetroxide catalyzed oxidations of organic compounds. J. Org. Chem..

[11] Piccialli V., Cavallo N. (2001). Improved RuO_4_-catalysed oxidative cyclisation of geraniol-type 1,5-dienes to *cis*-2,5-bis(hydroxymethyl)tetrahydrofuranyldiols. Tetrahedron Lett..

[12] Albarella L., Musumeci D., Sica D. (2001). Reactions of 1,5-dienes with ruthenium tetraoxide: Stereoselective synthesis of tetrahydrofurandiols. Eur. J. Org. Chem..

[13] Roth S., Göhler S., Cheng H., Stark C.B.W. (2005). A highly efficient procedure for ruthenium tetroxide catalyzed oxidative cyclizations of 1,5-dienes. Eur. J. Org. Chem..

[14] Göhler S., Cheng H., Stark C.B.W. (2007). Catalytic diastereo- and positionselective oxidative mono-cyclization of 1,5,9-trienes and polyenes. Org. Biomol. Chem..

[15] Göhler S., Roth S., Cheng H., Göksel H., Rupp A., Haustedt L.O., Stark C.B.W. (2007). Multigram synthesis of diastereomerically pure tetrahydrofuran-diols. Synthesis.

[16] de Champdoré M., Lasalvia M., Piccialli V. (1998). OsO_4_-catalyzed oxidative cyclization of geranyl and neryl acetate to *cis*-2,5-bis(hydroxymethyl)tetrahydrofurans. Tetrahedron Lett..

[17] Donohoe T.J., Winter J.J.G., Helliwell M., Stemp G. (2001). Hydrogen bonding control in the oxidative cyclisation of 1,5-dienes. Tetrahedron Lett..

[18] Donohoe T.J, Butterworth S. (2003). A general oxidative cyclization of 1,5-dienes using catalytic osmium tetroxide. Angew. Chem. Int. Ed..

[19] Klein E., Rojahn W. (1965). Oxidation of olefins by potassium permanganate. Oxygen-labeling experiments and mechanism of the oxidation of 1,5-hexadiene. Evidence for a manganese intermediate with coordination number greater than four. Tetrahedron.

[20] Baldwin J.E., Crossley M.J., Lehtonen E.-M.M. (1979). Stereospecificity of oxidative cycloaddition reactions of 1,5-dienes. J. Chem. Soc., Chem. Commun..

[21] Walba D.M., Wand M.D., Wilkes M.C. (1979). Stereochemistry of the permanganate oxidation of 1,5-dienes. J. Am. Chem. Soc..

[22] Walba D.M., Edwards P.D. (1980). Total synthesis of ionophores the monensin BC-rings via permanganate promoted stereospecific oxidative cyclization. Tetrahedron Lett..

[23] Spino C., Weiler L. (1987). A stereoselective synthesis of the tetrahydrofuran unit in ionomycin. Tetrahedron Lett..

[24] Walba D.M., Przybyla C.A., Walker C.B. J. (1990). Total synthesis of ionophores. 6. Asymmetric induction in the permanganate-promoted oxidative cyclization of 1,5-dienes. J. Am. Chem. Soc..

[25] Kocienskyi P.J., Brown R.C.D., Pommier A., Procter M., Schmidt B. (1998). Synthesis of salinomycin. J. Chem. Soc., Perkin Trans.1.

[26] Brown R.C.D., Hughes R.M., Keily J., Kenney A. (2000). Diastereoselective synthesis of tetrahydrofuran-containing fragments by the permanganate oxidation of 1,5,9-trienes. Chem. Commun..

[27] Brown R.C.D., Keily J. F. (2001). Asymmetric permanganate-promoted oxidative cyclization of 1,5-dienes by using chiral phase-transfer catalysis. Angew. Chem. Int. Ed..

[28] Towne T.B., McDonald F.E. (1997). *Syn*-oxidative polycyclizations of hydroxypolyenes: highly stereoselective and potentially biomimetic syntheses of *all*-*trans*-polytetrahydrofurans. J. Am. Chem. Soc..

[29] Morimoto Y., Iwai T. (1998). Highly diastereoselective cyclizations of bishomoallylic tertiary alcohols promoted by rhenium(VII) oxide. Critical steric versus chelation effects in alkoxyrhenium intermediates. J. Am. Chem. Soc..

[30] Sinha S.C., Keinan E., Sinha S.C. (1998). Rules of Stereoselectivity in Tandem Oxidative Polycyclization Reaction with Rhenium(VII) Oxides. J. Am. Chem. Soc..

[31] Keinan E., Sinha S.C. (2002). Oxidative polycyclizations with rhenium(VII) oxides. Pure Appl. Chem..

[32] Piancatelli G., Scettri A., D’Auria M. (1982). Pyridinium chlorochromate: a versatile oxidant in organic synthesis. Synthesis.

[33] Piccialli V., Zaccaria S., Oliviero G., D’Errico S., D’Atri V., Borbone N. (2011). Pyridinium chlorochromate-mediated oxidation of mono- and poly-tetrahydrofurans. Disclosure of novel oxidative pathways. Tetrahedron.

[34] Brimble M.A., Edmonds M.K. (1995). Synthesis of bis-2,5-linked tetrahydrofurans via iodoetherification. Tetrahedron.

[35] Nakata T., Nomura S., Matsukura H., Masamichi M. (1996). Stereoselective synthesis of the C- and CD-ring systems of hemibrevetoxin B. Tetrahedron Lett..

[36] Dickie D.A., Abeysekera D., McKenzie I.D., Jenkins H.A., Clyburne J.A.C. (2003). Crystallographic studies on substituted m-terphenyls: identification of weak [CH3··I] interactions. Cryst. Eng..

[37] Blessing R.H. (1995). An empirical correction for absorption anisotropy. Acta Crystallogr..

[38] Altomare A., Burla M.C., Cavalli M., Cascarano G.L., Giacovazzo C., Guagliardi A., Moliterni G.G., Polidori G., Spagna R. (1999). SIR97: a new tool for crystal structure determination and refinement. J. Appl. Crystallogr..

[39] Sheldrick G.M. (2008). A short history of SHELX. Acta Crystallogr..

[40] Farrugia L.J. (1999). WinGX suite for small-molecule single-crystal crystallography. J. Appl. Crystallogr..

[41] Cremer D., Pople J.A. (1975). General definition of ring puckering coordinates. J. Am. Chem. Soc..

[42] Nardelli M. (1995). PARST95 – an update to PARST: a system of Fortran routines for calculating molecular structure parameters from the results of crystal structure analyses. J. Appl. Crystallogr..

[43] Macrae C.F., Bruno I.J., Chisholm J.A., Edgington P.R., McCabe P., Pidcock E., Rodriguez-Monge L., Taylor R., van de Streek J., Wood P.A. (2008). Mercury CSD 2.0 – new features for the visualization and investigation of crystal structures. J. Appl. Cryst..

[44] Farrugia L.J. (1997). ORTEP-3 for Windows- a version of ORTEP-III with a graphical user interface (GUI). J. Appl. Cryst..

